# Personalized Approaches for the Prevention and Treatment of Breast Cancer

**DOI:** 10.3390/jpm12081201

**Published:** 2022-07-23

**Authors:** Hermann Nabi

**Affiliations:** 1 Oncology Division, CHU de Québec-Université Laval Research Center, Quebec City, QC G1S 4L8, Canada; hermann.nabi@crchudequebec.ulaval.ca; Tel.: +1-418-682-7511 (ext. 82800); 2 Université Laval Cancer Research Center (CRC), Université Laval, Quebec City, QC G1S 4L8, Canada; 3 Department of Social and Preventive Medicine, Faculty of Medicine, Université Laval, Quebec City, QC G1S 4L8, Canada

Breast cancer (BC) remains a major public health issue worldwide. According to recent estimates from the International Agency for Research on Cancer (IARC), female BC has become the most commonly diagnosed cancer type in the world, with 2.3 million cases diagnosed in 2020, exceeding the number of new cases of lung cancer for the first time [1]. Thanks to current advances in our understanding of cancer biology, BC is recognized as more than one disease. Indeed, BC presents as several distinct molecular subtypes, determined mainly by the presence and expression of biomarkers, such as hormone receptors (estrogen receptor (ER) and progesterone receptor (PR)) and human epidermal growth factor receptor (HER)-2, and, increasingly, by the genetic profile of the tumor [2,3,4]. Generally, BC is now classified into four major molecular subtypes: luminal A (ER-positive [ER+] and/or PR-positive [PR+], HER2− negative [HER2−], luminal B (ER+ and/or PR+, HER2+), triple-negative or basal-like (ER−, PR−, HER2−), and HER2-enriched (ER−, PR−, and HER2+) [5,6].

This molecular subtyping has been instrumental in the development of tailored therapeutic strategies for patients with BC, including endocrine and targeted therapies [7], and, maybe soon, immunotherapy [8]. However, primary or acquired resistances to these therapies are a major clinical obstacle, with a significant proportion of patients experiencing disease progression and death while taking these medications [9,10,11]. Notably, the reasons why some BC patients do not respond to endocrine or targeted therapies, and the factors that determine this response, are not well known. While progress has been made in the understanding of the underlying mechanisms of resistance to endocrine and targeted therapies [12,13,14], several potential lifestyle, clinical, and molecular determinants of response to these therapies remain insufficiently investigated. Identifying predictors of responsiveness to these therapies would provide insights to spare some women unnecessary toxicities and promote a selection of alternative treatment strategies for patients with resistant tumors.

Progress in genomics and the advent of new sequencing and digital-imaging technologies have also paved the way for applying personalized approaches to the early detection and/or prevention of breast cancer, even though their implementation at a population level faces considerable ethical, legal, social, and organizational challenges [15,16]. There are several reasons for supporting proposals for more personalized approaches to BC early detection and prevention, treatment, and follow-up. First, it is now possible to stratify healthy individuals as a function of their personal risk of BC using genetic and/or environmental risk factors and, subsequently tailor preventive recommendations [17]. Moving from a “one size fit all” prevention strategy to a more “personalized or stratified” strategy holds the prospect of achieving targeted and effective preventive interventions for women who are more likely to benefit from them. Second, patients, but also healthy individuals, are increasingly becoming more involved in health-related decision making and taking responsibility for their health according to their values, needs, and preferences [18,19]. In fact, the existing socioeconomic disparities in BC risk, risk factors, and outcomes are a clear indication that individuals do not all have the same needs [20,21]. Intuitively, this suggests that universal prevention, treatment, and follow-up strategies for BC are likely to be ineffective in the longer term, particularly in reducing these inequalities.

This Special Issue of the *Journal of Personalized Medicine* focuses, therefore, on personalized approaches relevant to BC detection, diagnosis, treatment, and survivorship and encompasses twelve manuscripts that will be briefly summarized and discussed here.

Relevant to the early detection of BC through mammography screening, Brooks et al. [22] provide an overview of the PERSPECTIVE I&I (Personalized Risk Assessment for Prevention and Early Detection of Breast Cancer: Integration and Implementation) project, which aims to test the feasibility and acceptability of the implementation of a risk-stratified, rather than age-based, approach to BC screening within existing Canadian screening programs. This international, multidisciplinary project comprises four interconnected activities covering all major dimensions of research (i.e., basic, clinical, and population). In addition to the WISDOM study in the United States (US) [23] and MyPeBS in Europe and Israel [24], the PERSPECTIVE I&I project constitutes one of the major international initiatives studying better ways to use risk-based stratification to prevent consequential cancers [15]. By assessing health system organizational readiness, and considering social, ethical, legal, and economic issues related to BC risk prediction and communication, this project is expected to generate much-needed evidence to support the implementation of a more personalized BC-screening approach.

The successful implementation of a personalized BC-screening approach within healthcare systems requires the buy-in of the most important stakeholder groups, including the women themselves. This important aspect is, in great part, covered by Mbuya-Bienge et al. [25] who gathered Canadian women’s views regarding this new screening approach. Collecting data from more than 4200 women aged 30 to 69 years from four of the largest provinces in Canada (i.e., Alberta, British Colombia, Ontario, and Quebec), they found that risk-based BC screening seems to be acceptable to most Canadian women. They also found several characteristics of women, including educational level, family income, ethnicity, and perceived risk of BC to have an influence on their views regarding the risk-stratified BC screening. Future studies might consider studying the profiles of women who exhibit favorable and unfavorable views in order to adapt communication strategies.

Using the same dataset, Alarie et al. [26] sought to examine women’s knowledge of the legislative context governing genetic discrimination (GD) and assess their concerns about the possible use of BC-risk-level information by insurers and employers. They found that Canadian women had limited knowledge of the regulatory framework related to GD. In addition, a third of them reported many concerns regarding the use of BC-risk-level information by insurers and employers. This suggests that education regarding policies governing GD and the existing legal protections is needed in the prospect of the implementation of a personalized BC-screening approach.

Since healthcare professionals (HPs) are expected to play a crucial role in the implementation of this approach, their views are also important to document. Blouin-Bougie et al. [27] undertook this work by conducting a qualitative study to explore HPs’ perceptions regarding the implementation of this screening approach in the province of Quebec, Canada. In general, they found that most participants reported positive attitudes towards the approach and agreed on its potential to improve the effectiveness of the BC-screening program. Nevertheless, their participants were concerned about the practicalities of the implementation of the approach, including the determination of the eligible populations, the method to reach them, the roles and responsibilities of clinicians, and the organization of the service delivery. Future studies assessing organizational readiness to implement a risk-stratified BC screening in existing health care systems would be needed [28]. In addition, gap analyses might shed light on the resources and interventions needed to potentiate the implementation of such an approach [29].

The implementation of a personalized BC-screening approach also poses ethical, legal, and social challenges. This is the perspective offered by Knoppers et al. [30]. In the first part of their review, they discussed the socio-ethical implications of risk stratification as a method to improve the benefit–arm balance of health-related interventions, including screening. Secondly, they elaborated on the regulations’ implications of polygenic risk scores (PRS) which have grown exponentially given the advances in genome-wide association studies (GWAS) [31]. They concluded by calling for legislators and regulators to provide clarification regarding issues such as the collection, storage, use, and sharing of datasets developed for the purpose of improving screening strategies and healthcare delivery.

In connection with socio-ethical issues related to genetic information, Hawranek et al. [32] conducted a focus group study on the perceptions of genetic-risk disclosure in members of the public in Sweden. Overall, they found that most participants reported a will to share genetic-risk information that can benefit others. However, mixed feelings were observed about the modalities of the disclosure of such information. Their results illustrate the complexity and even the dilemmas surrounding disclosure and highlight preferences on risk disclosure from the perspective of unaffected members of the public [33]. HPs’ perspectives on this issue are worth being investigated in future studies.

Regarding medical imaging for BC tumor detection, Chan et al. [34] explored automatic BC tumor detection by magnetic resonance (MR) diffusion-related technologies, such as intravoxel incoherent motion diffusion-weighted imaging (IVIM-DWI). To do so, they compared four methods that were found successful in detecting tumors for all the patients studied, even though one was found to be faster and the other displayed better predictive performance. They believe that these unsupervised tumor-detection methods have the advantage to potentially eliminate operator variability, which may lead to precise diagnosis. In the same vein, Costantini et al. [35] reported a case study of axillary-lymph-node metastases of occult breast cancer (CUPAx), an unusual condition that represents both a diagnostic and therapeutic challenge. Using contrast-enhanced spectral mammography (CESM), a new breast-imaging technique, they demonstrated a potential for the identification of occult BC in a CUPAx setting. Although replication in future studies is required, these findings highlight the potential of new breast-imaging approaches to help further personalize BC diagnosis and treatment strategies.

BC treatment has been a privileged area of application in precision medicine and it has undergone major development over the last two decades. Burguin et al. [36] conducted a comprehensive review to summarize current BC treatments and explore new, personalized treatment strategies and their associated challenges. This is an important piece of work since it provides a complete overview of the complexity of treatment options for BC which are constantly evolving, with a large number of ongoing clinical trials on emerging therapies. It is clear from this review that although proofs of efficacy of contemporary, personalized BC therapies are cumulated, not all women can benefit from them. Understanding, therefore, the underlying mechanisms of resistance to these therapies is certainly a good strategy to further develop novel treatments for BC.

Recently, cannabinoid receptors (CBR) appeared as potential therapeutic targets for BC [37], even though their role in BC survival remains to be elucidated. This was investigated by Morin-Buote et al. [38], who examined the associations of breast tumor expression of two types of CBR with prognostic factors and BC survival using data from a prospective cohort of 522 women diagnosed with invasive BC. They found CBR expression to be associated with several important clinicopathological prognostic risk factors, even though heterogeneity in tumors was observed. There was no evidence of association between markers of expression of CBR and survival outcomes, even though a lack of statistical power might explain this lack of association. Similarly, Zhang et al. [39] examined the effect of adjuvant whole-breast radiotherapy (WBRT) on clinical outcomes in women with left-side breast invasive ductal carcinoma and heart failure with reduced ejection fraction. They found adjuvant WBRT to be associated with a decreased risk of all-cause mortality, locoregional recurrence, and distant metastasis. Although these findings might help to further personalize the medical management of BC with IDC and HFrEF, replication in larger and diverse samples would be necessary. Finally, Gagnet et al. [40] used data from a real-world, retrospective cohort study to identify the clinicopathological factors associated with Oncotype DX (ODX) 21-gene recurrence score (RS), a gene-expression-profiling score used in clinical practice to predict the risk of recurrence and support treatment planning for women with early stage BC. They found that histologic grade and progesterone receptor (PR) status are predictive factors for intermediate or high RS. Given the cost of the test and the turnaround time to receive results, considering these clinicopathological factors could spare women the need to get such a test before the beginning of a possible adjuvant therapy. Moving forward, the development and validation of accurate prediction models for ODX RS based on relevant clinicopathological factors would be needed in order to identify patients who should undergo an adjuvant chemotherapy treatment.

To conclude, the manuscripts outlined in this Special Issue depict how personalized approaches can be integrated throughout BC control trajectories. The only missing dimension lies in BC survivorship. Due to a wide spectrum of varying health burdens and needs, there is a growing demand for personalized follow-up care after BC treatments [41,42]. Future studies are needed to test the acceptability and feasibility of BC follow-up tailored to patients’ risks and needs.
